# The Interactive Effect of *SIRT1* Promoter Region Polymorphism on Type 2 Diabetes Susceptibility in the North Indian Population

**DOI:** 10.1371/journal.pone.0048621

**Published:** 2012-11-01

**Authors:** Ekta Rai, Swarkar Sharma, Surabhi Kaul, Kamal Jain, Kawaljit Matharoo, Amarjit S. Bhanwer, Rameshwar N. K. Bamezai

**Affiliations:** 1 National Centre of Applied Human Genetics, School of Life Sciences, Jawaharlal Nehru University, New Delhi, India; 2 Department of Immunology, UT Southwestern Medical Center, Dallas, Texas, United States of America; 3 Department of Research, Texas Scottish Rite Hospital, Dallas, Texas, United States of America; 4 Department of Human Genetics, Guru Nanak Dev University, Amritsar, Punjab, India; Central China Normal University, China

## Abstract

Our previous studies have implicated genes mainly involved in the activity of pancreatic β cells in type 2 diabetes (T2D) susceptibility in the North Indian population. Recent literature on the role of *SIRT1* as a potential master switch modulating insulin secretion and regulating gene expression in pancreatic β cells has warranted an evaluation of *SIRT1* promoter region polymorphisms in the North Indian population, which is the main focus of the present study. 1542 samples (692 T2D patients and 850 controls) were sequenced for the 1.46 kb region upstream the translation start site of the *SIRT1* gene. We performed a functional characterization of the *SIRT1* promoter region polymorphisms using luciferase assay and observed a single-nucleotide polymorphism (SNP), rs12778366, in association with SIRT1 expression. We propose that TT, the high-expressing genotype of SNP rs12778366 in the *SIRT1* promoter region and present in >80% of the North Indian population, was favored under conditions of feast-famine cycles in evolution, which has turned out to be a cause of concern in the present sedentary lifestyle under *ad libitum* conditions. Case-control association analysis did not implicate rs12778366 in T2DM per se in the studied population. However, our earlier reported risk genotype combinations of mt-*ND3*, *PGC1α*, and *UCP2*-866, when compared with the protective genotype combinations, in the background of the high-expressing TT genotype of *SIRT1* SNP rs12778366, showed a very high additive risk [corrected odd ratio (OR) = 8.91; p = 6.5×10^−11^]. The risk level was considerably low in the genotype backgrounds of TX (OR = 6.68; p = 2.71×10^−12^) and CX (OR = 3.74; p = 4.0×10^−3^). In addition, we screened other reported T2D-associated polymorphisms: *PIK3R1* rs3730089, *IRS1* rs1801278, and *PPP1R3* rs1799999, which did not show any significant association in North Indian population. The present paper emphasizes the importance of gene interactions in the biological pathways of T2D, a complex lifestyle disease.

## Introduction

The tendency to develop diabetes was proposed to be a result of metabolic derangements in the functioning of genes that evolved as ‘thrifty’ [1] to regulate an efficient utilisation of fuel stores during feast and famine cycles. This is one of the most prominent hypotheses explaining genetic predisposition to type 2 diabetes (T2D). Further, the low level of insulin secretion or high insulin resistance was suggested to divert more energy to the insulin-independent tissues (brain and red blood cells) and less to the insulin-dependent tissues (skeletal muscles, liver, fat cells, and abdominal viscera) [2]. It was proposed that during starvation, low insulin level/insulin resistance makes more glucose available to the brain [3], which, due to a recent hypothesis, was further favored due to a shift in balance from a relatively muscle-dependent lifestyle to a more brain-dependent one [4], an evolutionary adaptation. Many genes have been proposed to play a role in this evolution [5]–[8]. According to recent literature [9]–[11], human sirtuins, especially SIRT1, are apparently involved and have a functional role in the process.

The human sirtuin SIRT1, a NAD^+^ histone deacetylase, is regulated by stress and nutritional status [12], regulating many transcription factors that modulate endocrine signalling and cellular metabolism in different physiological contexts. The role of SIRT1 has been demonstrated in nutrient-sensing and insulin-signalling pathways, as well as in the regulation of stress responses that determine cell survival, apoptosis, and proliferation [13]. For glucose homeostasis, in part by pancreatic β cells [14], SIRT1 is proposed to act as a potential master switch in β cell functions [15], in the modulation of insulin secretion [16], [17], and in the regulation of the activity of transcription factors and transcription co-regulators, which are implicated in the regulation of gene expression in pancreatic β cells [15]. SIRT1 regulates insulin secretion by repressing uncoupling protein 2 (UCP2) [16]. It affects glucose metabolism in liver cells by activating the transcription co-activator PGC1*α* with a subsequent expression of gluconeogenic genes and a repression of glycolytic genes. [18]. During fasting or calorie restriction, SIRT1 is involved in regulating pathways [13], [19] differentially in tissues, up-regulating in liver (increased glucose output through gluconeogenesis) and white adipose tissue (free fatty acid release) and down-regulating in pancreatic β cells (less insulin secretion) [20]. This differential activity favors the direction of glucose output mainly towards the brain. However, under *ad libitum* (or freely fed) conditions, SIRT1 expression is increased in pancreatic β cells, which induces an enhanced and efficient glucose-stimulated insulin secretion (GSIS) [15], [17] by repressing UCP2 [16].

Our studies on T2D in North India [21]–[23] suggest the interactive role of candidate gene polymorphisms in mitochondrial NADH dehydrogenase (mt-ND3) 10398G>A, peroxisome-proliferator-activated receptor co-activator-1 (PGC1*α*) (p.Thr394Thr or p.Gly482Ser), and UCP2-866G>A, mainly involved in the activity of pancreatic β cells, affecting GSIS. The key role of SIRT1 expression in pancreatic β cell functions provided us the reason to assess further the possible interactive role of the *SIRT1* promoter region. *SIRT1* has a potential to act as a ‘thrifty gene’ in Asian populations, mainly Indians, who have fewer cases of obesity but a higher prevalence of T2D at younger ages [24], probably due to the pronounced dysfunction in early insulin secretion in relation to insulin resistance [25]. The present study also evaluated other polymorphisms reported to be associated with T2D: phosphatidylinositol 3-kinase regulatory subunit (PIK3R1) p.Met326Ile (rs3730089), insulin receptor substrate 1 (IRS1) p.Gly971Arg (rs1801278), and protein phosphatase 1 regulatory subunit 3 (PPP1R3) p.Asp905Tyr (rs1799999) in the candidate genes known as the key nodes of cell signaling downstream of insulin and accounting for insulin resistance.

## Materials and Methods

In this study, a total of 1542 well-characterised samples (692 T2DM cases and 850 healthy controls) belonging to an Indo-European linguistic group from North India (Punjab with Jammu and Kashmir) were analysed. T2DM diagnosis was made in accordance with the criteria of World Health Organization and considering fasting glucose level of >126 mg/dl. Details of sample collection, along with inclusion and exclusion criteria are provided in our previous studies [21]–[23]. Clinical details and other information about the studied samples are tabulated in Table S1.

### Ethics statement

A written informed consent was obtained from all the participants. The data were analysed anonymously, and the study was approved by the ethical committee of Jawaharlal Nehru University.

### Genotyping of the polymorphisms

**Figure 1:**
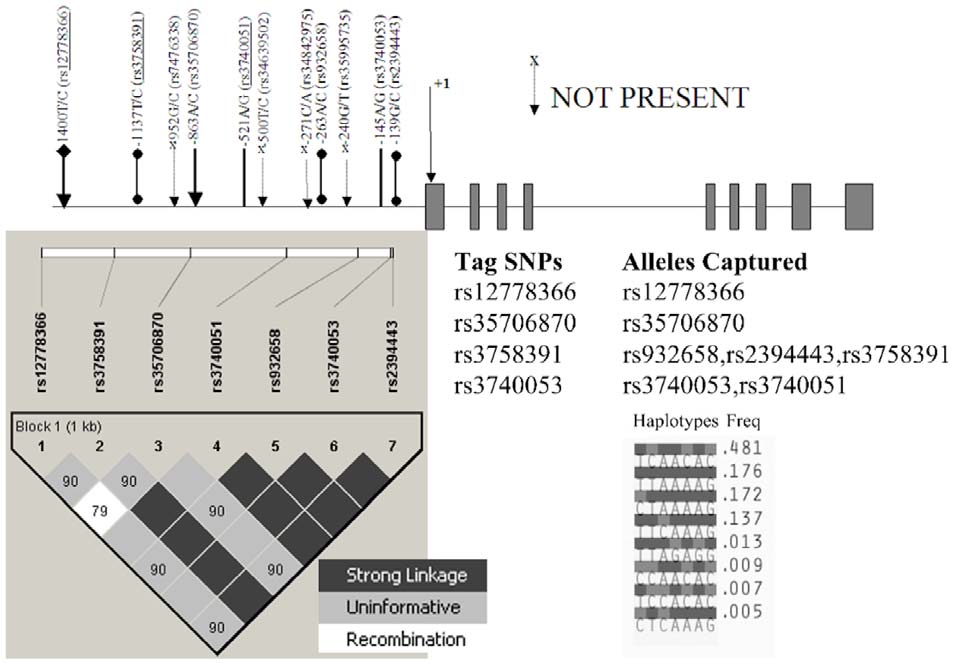
Promoter region of *SIRT1* gene. Figure provides the details of the 11 promoter polymorphisms of the *SIRT1* gene spanned through 1.46 kb region upstream the translation start site. 4 SNPs marked by ‘X’ did not show polymorphism in the studied population. D′ values are plotted as a graph to show linkage disequilibrium between the 7 observed polymorphic markers. Frequencies of the observed haplotypes and details of the picked Tag SNPs and respective alleles captured among the 7 polymorphic markers are also provided.

Genomic DNA was isolated from peripheral blood through a routine protocol used in laboratories. We sequenced 1.46 kb upstream of the translation start site of the *SIRT1* gene and observed eleven SNPs (rs12778366, rs3758391, rs7476338, rs35706870, rs3740051, rs34639502, rs34842975, rs932658, rs35995735, rs3740053, and rs2394443). However, only 7 SNPs (rs12778366, rs3758391, rs35706870, rs3740051, rs932658, rs3740053, and rs2394443) were found polymorphic in the North Indian population (Table S2 and Fig. 1). For this purpose, oligos were designed using PRIMER 3 software [26]. A primer pair (forward 5′ ACGCAACCAAAGATGGTTTT 3′ and reverse 5′ CTTCCAACTGCCTCTCTGG 3′) was used to amplify the whole region. The total PCR reaction mix made was 12.5 µl, containing ∼80 ng of template DNA, 6.25 pmoles of each primer, 300 µM of dNTPs, 1.5 mM of MgCl_2_, 1X reaction buffer, and 0.4 units of the *Taq* pol enzyme (Bangalore Genei, India). The cycling conditions were as follows: denaturation at 95°C for 1 min, followed by annealing at 62°C for 1 min, and then extension at 72°C for 1.5 min, repeated for 32 cycles followed by a final extension step at 72°C for 10 min. PCR products were initially checked in 1.5% agarose gel, sequenced using ABI Prism 3100–Avant Genetic Analyzer (Applied Biosystems, USA), and analysed in SeqScape software v2.1.1 (Applied Biosystems). Two internal primers (Forward 5′ TGCACGTGAGAAAACTGAGG 3′ and Reverse 5′ ACCTTTGACGTGGAGGTTTG 3′) were also used along with external primers for a complete sequencing without any gap. The genotypic data of mt-*ND3* (rs2853826), *PGC1α* variants (rs2970847 and rs8192678), and *UCP2* (rs659366) for the samples were retrieved from our earlier publication [21]. Previously defined PCR and restriction digestion methodologies from literature were used to screen other candidate gene polymorphisms: *PIK3R1* p.Met326Ile (rs3730089), *IRS1* p.Gly971Arg (rs1801278), and *PPP1R3* p.Asp905Tyr (rs1799999). For quality control and to confirm restriction digestions, direct sequencing was performed to screen these polymorphisms in 250 randomly selected samples, which showed 100% concordance, suggesting no error in restriction digestion methods.

### Statistical analyses

The Hardy–Weinberg equilibrium was tested at each variant locus in a contingency table of observed versus predicted genotype frequencies using the chi-square test. Categorical variables were compared using the chi-square (χ^2^) test. Continuous data were shown as mean±SD. Logistic regression analysis was used to determine the independent and interactive associations of the allelic and genotypic status of the studied polymorphisms with T2DM. Corrections for age, sex, body mass index (BMI) and center of sample collection were also performed. Bonferroni correction was adopted for multiple hypothesis testing.

Interaction between the various genotypes (different genes) was established mainly by binary logistic regression in statistical package for social science program (SPSS version 13.0; SPSS, Chicago, IL). The risk and protective genotype combinations for mt-*ND3* rs2853826, *PGC1α* rs8192678, and *UCP2* rs659366GG were first established (Table S2), also reported in our previous study [21]. Further, considering SIRT1 as an upstream candidate, individuals with the *SIRT1* rs12778366 (-1400) genotypic backgrounds (TT, TX, CX) were categorized and risk and protective genotypic distributions of downstream candidate genes: mt-*ND3* rs2853826, *PGC1α* rs8192678, and *UCP2* rs659366GG, were estimated in those backgrounds individually and collectively. These distributions in cases and controls were used to estimate the odd ratios (ORs) at 95% confidence interval (CI) and the respective *p* values. Empirical p values were also estimated for all risk Versus protective genotypes of mt-ND3 rs2853826, PGC1*α* rs8192678 and UCP2 rs659366 in the *SIRT1* rs12778366 (-1400) genotypic backgrounds (TT, TX, CX) by logistic regression using PLINK [27] adaptive permutations (maximum1,000,000 permutations). The linkage disequilibria between variants within the 1.4 kb region upstream the translation start site of the SIRT1 gene, the detection of tag single-nucleotide polymorphisms (SNPs) in the region, and haplotypic disease association were done using Haploview 4.0 software (www.hapmap.org). The statistical power of this study for each polymorphism was estimated by using PS software version 2.1.31 [28]. The statistical analyses were mainly performed using the statistical package for social science program (SPSS version 13.0; SPSS, Chicago, IL).

### Functional characterisation of the SIRT1 promoter region polymorphisms using Dual-Luciferase Assay

The distal region upstream the translation start site of the *SIRT1* gene, encompassing SNPs rs12778366, rs3758391, rs7476338, and rs35706870, was amplified from individuals with already genotyped and known genotypic backgrounds using the following primers: forward 5′ ACGCAACCAAAGATGGTTTT 3′ and reverse 5′ ACCTTTGACGTGGAGGTTTG 3′. The amplified products were cloned in a TOPO cloning vector (Invitrogen, Carisbad, CA). They were then digested with SacI and XhoI (New England Biolabs, USA) for cloning into pGL3-Promoter Vector (Promega, Madison, WI). pGL3-Promoter Vector contains an SV40 promoter upstream of the luciferase gene and helps identify enhancer elements. The same region was also cloned in the pGL3 basic vector (Promega, Madison, WI) to ascertain whether this region shows minimal promoter activity as this vector lacks eukaryotic promoter and enhancer sequences. Sequencing (ABI Prism 3100–Avant Genetic Analyzer, Applied Biosystems) was done to confirm the positive clones and the allelic status at the mentioned SNPs. The Dual-Luciferase (Firefly luciferase and Renilla luciferase) Reporter Assay System (Promega) was used to perform luciferase assays (in triplicates) for different allelic combinations of the mentioned polymorphisms. Reporter gene activities were corrected for background luminescence and then for differences in cell densities and transfection efficiencies by dividing firefly luciferase activity with Renilla luciferase activity. A detailed methodology for transient transfection of the human cervical carcinoma cell line (HeLa cells) and luciferase assays is provided elsewhere [29]. The proximal region to translation start site (spanned by primers: forward 5′ TGCACGTGAGAAAACTGAGG 3′ and reverse 5′ CTTCCAACTGCCTCTCTGG 3′) was cloned in pGL3 basic vector (Promega, Madison, WI) but the variants in the region did not show significant difference in Luciferase activity (data not shown).

## Results

### Genotypic distribution of the *SIRT1* promoter region polymorphisms

Out of the 11 genotyped SNPs of the promoter region of the *SIRT1* gene, only 7 SNPs (rs12778366, rs3758391, rs35706870, rs3740051, rs932658, rs3740053, and rs2394443) were found polymorphic in the North Indian population (Table S2 and Fig. 1). All the polymorphisms were observed with high LD (D′≥0.79) (Fig. 1). Haplotypes in 1542 individuals were analysed using Haploview software. A total of eight haplotypes were observed (Table S3 and Fig. 1). The haplotype ‘TCAACAC’ at the seven polymorphic SNPs (rs12778366, rs3758391, rs35706870, rs3740051, rs932658, rs3740053, and rs2394443) was observed with the highest frequency of 0.481 in the North Indian population. None of the haplotypes showed any significant difference in frequency distribution among cases and controls (Table S3). Four tag SNPs (Fig. 1) ascertained 100% of alleles (in the 1.46 kb region upstream the translation start site of the *SIRT1* gene) with a mean *r* ^2^ of 1.0 in the studied population group. rs12778366 and rs35706870 were uninformative for any other SNPs, whereas rs3758391 could capture alleles at rs932658, rs2394443, and rs3758391; rs3740053 was informative to capture alleles at rs3740053 and rs3740051 (Fig. 1).

### Functional characterisation of the *SIRT1* promoter region polymorphisms

**Figure 2:**
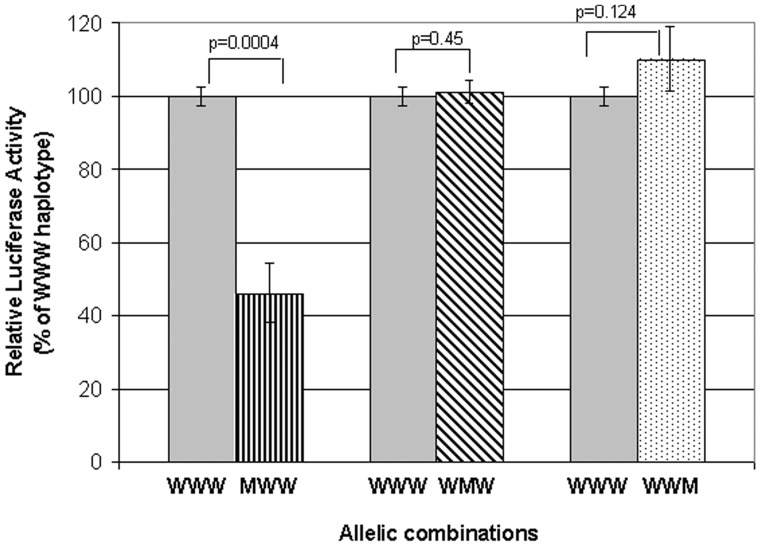
The observed relative luciferase activities. The relative activities of the haplotypes (with alleles at rs12778366 T>C, rs3758391 T>C and rs35706870 A>C, respectively) are shown. Combinations of: mutant (M) at rs12778366 with rest wild (W) i.e., MWW = ‘CTA’; mutant at rs3758391 and rest wild i.e., WMW = ‘TCA’ ; and mutant at rs35706870 with rest wild i.e., WWM = ‘TTC’ were compared against all wild i.e., WWW = ‘TTA’.

Luciferase assays were performed to explore the possible role of *SIRT1* promoter region polymorphisms on the expression profile of the *SIRT1* gene (Fig. 2). We simultaneously tested alleles at rs12778366 T>C, rs3758391 T>C, and rs35706870 A>C as haplotypes, and the following combinations were studied: all wild ‘TTA’ (WWW), mutant at rs12778366; others wild ‘CTA’ (MWW), mutant at rs3758391; rest wild ‘TCA’ (WMW), mutant at rs35706870; and others wild ‘TTC’ (WWM). The observed relative luciferase activities of the haplotypes were compared with those of all wild haplotypes (WWW). It was observed that only MWW haplotypes showed a highly significant (*p* = 0.0004) reduction (>50%) in the expression of the luciferase reporter gene (Fig. 2). A similar region with haplotype (WWW) was also cloned in the pGL3 basic vector, which lacks eukaryotic promoter and enhancer sequences. This clone did not show any luciferase activity, asserting that this region does not contain a minimal promoter (activity) of the *SIRT1* gene, hence showing only an enhancer activity.

### Independent and interactive analyses of the polymorphisms

The re-assessment of the association of mt-ND3 rs2853826, PGC1*α* variants (rs2970847 and rs8192678), and UCP2 rs659366 polymorphisms with T2D (Table S2) in cases and controls, genotyped for polymorphisms in the proposed genes in the present study, showed similar significant associations, as reported earlier [21]. PIK3R1 rs3730089, IRS1 rs1801278, and PPP1R3 rs1799999 reported to be associated with T2D in literature did not show any association in the studied samples (Table S2).

For the interactive analysis, 1312 samples (603 cases and 709 controls), with complete genotypic information at all the studied SNPs, were chosen. Initially, genotypic combinations of any two genes were compared. In the independent analysis, none of the *SIRT1* polymorphisms/haplotypes *per se* showed a significant difference in distribution between cases and controls (Table S3). However, in the interactive analysis, the *SIRT1* rs12778366 (-1400) genotypic background (considering SIRT1 as an upstream candidate) appeared to show an effect on the ORs when analysed for mt-ND3 rs2853826 and UCP2 rs659366 genotypes (Table 1). As previously reported [21], when pooling and comparing all the three risk genotype combinations—mt-ND3 rs2853826 A, PGC1*α* rs8192678 XA, and UCP2 rs659366 GG—against respective protective genotype combinations, an additive interactive effect (corrected OR = 6.88, 4.30–10.97 at 95% CI; *p* = 6.18×10^−13^) was observed (Table 1). Interestingly, the additive risk increased when a similar comparison between risk and protective genotypes was assessed in the *SIRT1* rs12778366 TT genotype background (corrected OR = 8.91, 4.62–17.17 at 95% CI; *p* = 6.5×10^−11^), and this risk reduced in *SIRT1* rs12778366 TX (corrected OR = 6.68, 3.92–11.37 at 95% CI; *p* = 2.71×10^−12^) and CX (corrected OR = 3.74, 1.54–9.10 at 95% CI; *p* = 8.7×10^−4^) genotype backgrounds. In the CC background, this interaction could not be performed due to a very low sample size (Table 1).

**Table 1:** Interactive odds ratio estimation of mt-*ND3* rs2853826, *PGC1α* rs8192678, *UCP2* rs659366 genotypes in *SIRT1* rs12778366 genotype backgrounds.

n = 1312 samples (603 cases and 709 controls)					
		ORs in Genotype background of *SIRT1* rs12778366			
Gene polymorphism Tested	Independent OR (95% CI), p value	TT (n = 879)	TX (n = 1255)	CX (n = 433)	CC (n = 57)
mt-*ND3* rs2853826 A Vs G	1.48 (1.22–1.82), p = 1.0×10^−4^	1.47 (1.13–1.92), p = 4.0×10^−3^	1.38 (1.11–1.38), p = 4.0×10^−3^	NS	NS
*PGC1α* rs8192678 XA Vs GG	2.67 (2.13–3.33), p = 4.9×10^−12^	1.94(1.44–2.60), p = 1.18×10^−8^	2.12 (1.65–2.72), p = 3.65×10^−9^	2.18 (1.38–3.45), p = 8.0×10^−4^	NS
*UCP2* rs659366 GG Vs XA	1.45 (1.18–1.78), p = 4.68×10^−4^	1.75(1.34–2.29), p = 4.42×10^−5^	1.60 (1.28–2.0), p = 3.79×10^−5^	NS	NS
*mt-*ND3* rs2853826, *PGC1α* rs8192678 and *UCP2* rs659366 all risk (A, XA and GG respectively) Vs all protective (G, GG and XA respectively) [empirical p values]	6.88(4.30–10.97)^a^,p = 6.18×10^−13^ [p^e^ = 1.0×10^−6^]	8.91 (4.62–17.17)^b^, p = 6.5×10^−11^ [p^e^ = 1.0×10^−6^]	6.68 (3.92–11.37)^c^, p = 2.71×10^−12^ [p^e^ = 1.0×10^−6^]	3.74(1.54–9.10)^d^, p = 4.0×10^−3^ [p^e^ = 8.7×10^−4^]	NA

P values and odds Ratios are estimated by binary logistic regression using age, gender, BMI and center of collection as covariates.

NS = Not significant, NA = Not available

* all risk genotypic combinations (mt-*ND3* rs2853826 A, *PGC1α* rs8192678 XA, *UCP2* rs659366 GG) were compared against all protective genotypic combinations (mt-*ND3* rs2853826 G, *PGC1α* rs8192678 GG, *UCP2* rs659366 XA). a = independent of *SIRT1* background (n = 292) and in the background *SIRT1* genotypes, b = TT (n = 185), c = TX (n = 276) d = CX (n = 107)

e = empirical p values were estimated by logistic regression using PLINK adaptive permutations (maximum1,000,000 permutations).

## Discussion

Complex diseases, such as T2D, have attained an epidemic proportion in recent years within Asia, especially in India. This has been attributed to the prevalent sedentary lifestyle and high-calorie diet that affect human physiology and the level of expression of the genes involved mainly in fuel metabolism. It is, therefore, pertinent to understand the molecular basis of T2D and find potential genes with specific genotype backgrounds, providing susceptibility to the disease.

SIRT1 is activated under a calorie-restriction regimen, which qualifies for a favorable selection under prevailing feast-famine conditions in evolution. SIRT1 has been suggested to regulate several downstream genes [13], [15], [20] to maintain glucose homeostasis in the body. Previous studies, including that of ours [21]–[23], suggested that the specific genotypes of these downstream genes with an additive interaction result in risks of developing T2D [21]. The present study shows that the high-expressing genotype ‘TT’ of the promoter region polymorphism (rs12778366), evident by the *in vitro* luciferase assay, may affect the expression profile of SIRT1. In this background, risk genotypes of mt-ND3 rs2853826, PGC1*α* rs8192678, and UCP2 rs659366 showed an enhanced effect on the risk of developing T2D.

SIRT1 affects glucose metabolism in liver cells by activating the transcription co-activator PGC1*α* with a subsequent expression of gluconeogenic genes and a repression of glycolytic genes. [18] Further, it has been implicated in differentially regulating pathways [13], [19] in tissues by its upregulation in the liver (increased glucose output by gluconeogenesis) and white adipose tissue (free fatty acid release) and by downregulation in pancreatic β cells (less insulin secretion) [20]. This differential activity results in the direction of the glucose output mainly toward the brain. However, under *ad libitum* conditions, SIRT1 expression is increased in pancreatic β cells [20], which induces enhanced and efficient GSIS [15], [17] by repressing UCP2 [16]. Thus, *SIRT1* could be a potential thrifty gene [1], and this hypothesis may find support in recent observations where *SIRT1* variants were reported to affect diabetes risk in interaction with prenatal exposure to famine [10]. We suggest that, in evolution, the TT genotype of *SIRT1* rs12778366 (-1400) was selected favorably. This further favored a low level of insulin secretion in β cells of pancreas under starvation to divert more energy to the insulin-independent tissues (brain and red blood cells) and less to the insulin-dependent tissues (skeletal muscles, liver, fat cells, and abdominal viscera) [2] or to make more glucose available to the brain [3] as an evolutionary adaptation due to the shift in balance from a relatively muscle-dependent lifestyle to a more brain-dependent one [4]. That is the reason one finds more than 80% frequency of the T allele in the population. However, this selection, which happened under conditions of starvation, has turned out to be a cause of concern because under *ad libitum* situations, the β cells of pancreas would be producing more insulin [20] in the TT genotype background of *SIRT1* rs12778366 and would create a cycle of hyperinsulinemia and hyperglycemia unless the efficient pancreatic system maintains glucose homeostasis. This homeostasis, however, seems to be unbalanced when the genotype conditions of mtDNA and the low availability of UCP2 encourage the mitochondria to produce more reactive oxygen species (ROS) and damage the β cells progressively over a period, thus developing into T2D with time. It is apparent from our results that the SNP rs12778366 TT background showed a high additive risk (corrected OR = 8.91, 4.62–17.17 at 95% CI; *p* = 6.5×10^−11^) when the reported risk versus protective genotype combinations in mt-ND3, PGC1*α*, and UCP2 variations were compared. A probable dose-dependent decrease in risk was observed when risk genotypes mt-*ND3* rs2853826 A, *PGC1α* rs8192678 XA, and *UCP2* rs659366 GG were evaluated in rs12778366 TX (corrected OR = 6.68, 3.92–11.37 at 95% CI; *p* = 2.71×10^−12^) and rs12778366 CX (corrected OR = 3.74, 1.54–9.10 at 95% CI; *p* = 8.7×10^−4^) backgrounds. In our previous [21]–[23] studies too, we have shown that mt-*ND3* rs2853826 A, *PGC1α* rs8192678 XA, and *UCP2* rs659366 GG provide risk towards T2DM individually and interactively which was explained by the functional role of the genes. Mt-*ND3* rs2853826 A has been associated with increased mitochondrial ROS production [30]. UCP2 has been reported to provide a protective effect against ROS, in particular superoxides of mitochondrial origin [31], [32]. *PGC1α* has been implicated in mitochondrial biogenesis, adaptive thermogenesis (reviewed in [33]), reported to play an important role in expression of UCP2 in pancreatic β cells [34] and involvement in induction of ROS-detoxifying enzymes [35]. It might indicate that relatively low producer genotypes of *SIRT1* rs12778366 (TX/CX/CC) could provide protection probably due to less suppression effect on UCP2, allowing the efficient removal of ROS from the β cells. However, this protective effect is probably not enough to avoid a complete risk provided by the risk genotypes mt-*ND3* rs2853826 A, *PGC1α* rs8192678 XA, and *UCP2* rs659366 GG, causing damage to pancreatic β cells.

To conclude, our study suggests the importance of understanding the ‘genotype mosaic’ context of risk-attributing variation in a gene and the nature of its interaction with other genes in a pathway. We propose that there would be several layers of different genotypes of known and unknown genes in the biological pathways of T2D. SIRT1 expression could be influenced by other functional variants in the gene. Hence, a thorough exploration of *SIRT1* gene variations in North Indian population and extension of present work in a larger data set is warranted for conclusions. Also, it would be interesting to see if these observations replicate in other population groups. Nevertheless, a study such as ours, apart from genome-wide association studies, helps in putting together the parts of a jigsaw puzzle one after the other and adds to the understanding of the phenomenology of the disease pathology in the context of evolutionary selection of genotypes in different population groups.

## Supporting Information

Table S1 **Clinical and other demographic details of cases and controls in the present study.**
(DOC)

Table S2 **Distribution of the studied SNPs in T2DM patients and controls of North India.**
(DOC)

Table S3 **Haplotypic frequency distribution of** ***SIRT1*** **promoter region polymorphisms in cases and controls from north Indian population.**
(DOC)
