# Supplementary material for: The Interactive Effect of SIRT1 Promoter Region Polymorphism on Type 2 Diabetes Susceptibility in the North Indian Population
Source: PLoS One. 2012 Nov 1;7(11):e48621. doi: 10.1371/journal.pone.0048621 (PMC3486794; doi:10.1371/journal.pone.0048621)
Supplement: Table S1 — Clinical and other demographic details of cases and controls in the present study. (DOC) [file pone.0048621.s001.doc]

Supplementary Table S1: Clinical and other demographic details of cases and controls in the present study.

| **Characteristics** | **Cases**  n=692 | **Controls**  n=850 |
| --- | --- | --- |
|  |  |  |
| Age (SD) | 53.78 (10.65) | 48.14 (10.21) |
| BMI (SD) | 25.77 (4.96) | 25.16 (4.86) |
| Waist-Hip ratio (SD) | 0.97 (0.082) | 0.94 (0.068) |
| Systolic Blood Pressure (SD) | 139.54 (21.75) | 132.5 (20.63) |
| Diastolic Blood Pressure(SD) | 88.78 (11.29) | 86.03 (11.57) |
| Fasting Sugar (SD) | 189.9 (81.48) |  |
| Random Sugar (SD) | 222.26 (82.31) | 102.1 (14.63) |
| Age of Onset (SD) | 46.48 (10.08) |  |
| Sex | F=47.38% M=52.62% | F=48.15% M=51.85% |
| Diet-Veg | 51.38% | 60.53% |
| Alcohol/Smoking | 13.45% | 9.27% |
| Insulin treatment | 5.45% |  |
| Family History | 35.96% |  |
