# Supplementary material for: The Interactive Effect of SIRT1 Promoter Region Polymorphism on Type 2 Diabetes Susceptibility in the North Indian Population
Source: PLoS One. 2012 Nov 1;7(11):e48621. doi: 10.1371/journal.pone.0048621 (PMC3486794; doi:10.1371/journal.pone.0048621)
Supplement: Table S2 — Distribution of the studied SNPs in T2DM patients and controls of North India. (DOC) [file pone.0048621.s002.doc]

Supplementary Table S2: Distribution of the studied SNPs in T2DM patients and controls of North India.

| **Gene** | **SNP ID** | **Polymorphism** |  | n | Alleles | |  | **Genotypes** | | |
| --- | --- | --- | --- | --- | --- | --- | --- | --- | --- | --- |
| SIRT1 | rs12778366 | -1400 T/C |  | n | **T** | C |  | **TT** | **TC** | **CC** |
|  |  |  | Cases | 692 | 0.827 | 0.173 |  | 0.691 | 0.273 | 0.036 |
|  |  |  | Controls | 850 | 0.802 | 0.198 |  | 0.648 | 0.308 | 0.044 |
|  |  |  |  |  | NS | |  | NS | | |
|  | rs3758391, rs932658, rs2394443 | -1137 T/C, -263 A/C, -139 G/C |  |  | **T-A-G** | **C-C-C** |  | **TT-AA-GG** | **TC-AC-GC** | **CC-CC-CC** |
|  |  |  | Cases | 692 | 0.496 | 0.504 |  | 0.25 | 0.491 | 0.259 |
|  |  |  | Controls | 850 | 0.509 | 0.491 |  | 0.28 | 0.459 | 0.261 |
|  |  |  |  |  | NS | |  | NS | | |
|  | rs35706870 | -863 A/C |  |  | **A** | **C** |  | **AA** | **AC** | **CC** |
|  |  |  | Cases | 692 | 0.848 | 0.152 |  | 0.724 | 0.249 | 0.027 |
|  |  |  | Controls | 850 | 0.853 | 0.147 |  | 0.738 | 0.231 | 0.032 |
|  |  |  |  |  | NS | |  | NS | | |
|  | rs3740051, rs3740053 | -521 A/G, -145 A/G |  |  | **A-A** | **G-G** |  | **AA-AA** | **AG-AG** | **GG-GG** |
|  |  |  | Cases | 692 | 0.986 | 0.014 |  | 0.982 | 0.006 | 0.012 |
|  |  |  | Controls | 850 | 0.989 | 0.011 |  | 0.986 | 0.006 | 0.008 |
|  |  |  |  |  | NS | |  | NS | | |
|  | rs35995735 (G/T), rs34842975 (C/A), rs34639502 (T/C), rs7476338 (G/C) | | Cases | 692 | **No polymorphic change (only GG-CC-TT-GG genotypes)** | | | | | |
|  |  |  | Controls | 850 |  |  |  |  |  |  |
| ***mt-ND3***  **10398** | rs2853826 | Ala114Thr (G/A) |  |  | **G** | **A** |  | **GG** | **GA** | **AA** |
|  |  |  | Cases | 692 | 0.45 | 0.55 |  | NA | NA | NA |
|  |  |  | Controls | 850 | 0.58 | 0.42 |  | NA | NA | NA |
|  |  |  |  |  | P=3.3 x 10^-7^ | |  | NS | | |
|  |  |  |  |  | OR=1.69 (1.38-2.07) | |  | NS | | |
| PGC1α | rs2970847 | Thr394Thr (G/A) |  |  | **G** | **A** |  | **GG** | **GA** | **AA** |
|  |  |  | Cases | 603 | 0.38 | 0.62 |  | 0.21 | 0.341 | 0.449 |
|  |  |  | Controls | 709 | 0.519 | 0.481 |  | 0.353 | 0.333 | 0.314 |
|  |  |  |  |  | P=1.1 x 10^-14^ | |  | p=6.35 x 10^-10^ | | |
|  |  |  |  |  | OR=1.76 (1.53-2.04) | |  | OR=2.06 (1.63-2.59) XA Vs GG | | |
|  | rs8192678 | Gly482Ser (G/A) |  |  | **G** | **A** |  | **GG** | **GA** | **AA** |
|  |  |  | Cases | 603 | 0.48 | 0.52 |  | 0.23 | 0.5 | 0.27 |
|  |  |  | Controls | 709 | 0.662 | 0.338 |  | 0.444 | 0.435 | 0.121 |
|  |  |  |  |  | P=3.37 x 10^-24^ | |  | P=1.83 x 10^-18^ | | |
|  |  |  |  |  | OR=2.12(1.83-2.45) | |  | OR=2.67 (2.14-3.34) XA Vs GG | | |
| UCP2 | rs659366 | -866 G/A |  |  | **G** | **A** |  | **GG** | **GA** | **AA** |
|  |  |  | Cases | 692 | 0.682 | 0.318 |  | 0.462 | 0.448 | 0.09 |
|  |  |  | Controls | 850 | 0.624 | 0.376 |  | 0.339 | 0.558 | 0.104 |
|  |  |  |  |  | p=6.25 x 10^-5^ | |  | p=6.68 x 10^-7^ | | |
|  |  |  |  |  | OR=0.74 (0.63-0.86) | |  | OR=0.59(0.48-0.73) XA Vs GG | | |
| PIK3R1 | rs3730089 | Met326Ile (G/T) |  |  | **G** | **T** |  | **GG** | **GT** | **TT** |
|  |  |  | Cases | 692 | 0.457 | 0.543 |  | 0.211 | 0.491 | 0.298 |
|  |  |  | Controls | 850 | 0.465 | 0.535 |  | 0.2 | 0.53 | 0.27 |
|  |  |  |  |  | NS | |  | NS | | |
| IRS1 | rs1801278 | Gly971 Arg (G/A) |  |  | **G** | **A** |  | **GG** | **GA** | **AA** |
|  |  |  | Cases | 692 | 0.906 | 0.094 |  | 0.831 | 0.15 | 0.019 |
|  |  |  | Controls | 850 | 0.909 | 0.091 |  | 0.827 | 0.165 | 0.008 |
|  |  |  |  |  | NS | |  | NS | | |
| PPP1R3 | rs1799999 | Asp905Tyr (G/T) |  |  | **G** | **T** |  | **GG** | **GT** | **TT** |
|  |  |  | Cases | 692 | 0.574 | 0.426 |  | 0.308 | 0.532 | 0.16 |
|  |  |  | Controls | 850 | 0.579 | 0.421 |  | 0.32 | 0.518 | 0.162 |
|  |  |  |  |  | NS | |  | NS | | |
