# Supplementary material for: The Interactive Effect of SIRT1 Promoter Region Polymorphism on Type 2 Diabetes Susceptibility in the North Indian Population
Source: PLoS One. 2012 Nov 1;7(11):e48621. doi: 10.1371/journal.pone.0048621 (PMC3486794; doi:10.1371/journal.pone.0048621)
Supplement: Table S3 — Haplotypic frequency distribution of SIRT1 promoter region polymorphisms in cases and controls from north Indian population. (DOC) [file pone.0048621.s003.doc]

Supplementary Table S3: Haplotypic frequency distribution of *SIRT1* promoter region polymorphisms in cases and controls from north Indian population.

| **Haplotype** | **Frequency** | | |  |
| --- | --- | --- | --- | --- |
|  | **Population** | **Cases** | **Controls** | **p value** |
|  |  |  |  |  |
| TCAACAC | 0.481 | 0.492 | 0.472 | 0.2653 |
|  |  |  |  |  |
| TTAAAAG | 0.176 | 0.172 | 0.179 | 0.5976 |
|  |  |  |  |  |
| CTAAAAG | 0.172 | 0.163 | 0.179 | 0.2422 |
|  |  |  |  |  |
| TTCAAAG | 0.137 | 0.143 | 0.132 | 0.404 |
|  |  |  |  |  |
| TTAGAGG | 0.013 | 0.014 | 0.011 | 0.4183 |
|  |  |  |  |  |
| CCAACAC | 0.009 | 0.006 | 0.011 | 0.154 |
|  |  |  |  |  |
| TCCACAC | 0.007 | 0.006 | 0.008 | 0.6 |
|  |  |  |  |  |
| CTCAAAG | 0.005 | 0.003 | 0.007 | 0.119 |

Haplotype consists of rs12778366, rs3758391, rs35706870, rs3740051, rs932658, rs3740053, rs2394443, respectively
